# TCEDN: A Lightweight Time-Context Enhanced Depression Detection Network

**DOI:** 10.3390/life14101313

**Published:** 2024-10-16

**Authors:** Keshan Yan, Shengfa Miao, Xin Jin, Yongkang Mu, Hongfeng Zheng, Yuling Tian, Puming Wang, Qian Yu, Da Hu

**Affiliations:** 1 School of Software, Yunnan University, Kunming 650000, China; 12022219123@mail.ynu.edu.cn (K.Y.); muyongkang@stu.ynu.edu.cn (Y.M.);; 2 Engineering Research Center of Cyberspace, Yunnan University, Kunming 650000, China; 3 Fengtu Technology (Shenzhen) Co., Ltd., Shenzhen 518057, China

**Keywords:** video depression detection, deep learning, 3D-CNN, ConvLSTM, attention

## Abstract

The automatic video recognition of depression is becoming increasingly important in clinical applications. However, traditional depression recognition models still face challenges in practical applications, such as high computational costs, the poor application effectiveness of facial movement features, and spatial feature degradation due to model stitching. To overcome these challenges, this work proposes a lightweight Time-Context Enhanced Depression Detection Network (TCEDN). We first use attention-weighted blocks to aggregate and enhance video frame-level features, easing the model’s computational workload. Next, by integrating the temporal and spatial changes of video raw features and facial movement features in a self-learning weight manner, we enhance the precision of depression detection. Finally, a fusion network of 3-Dimensional Convolutional Neural Network (3D-CNN) and Convolutional Long Short-Term Memory Network (ConvLSTM) is constructed to minimize spatial feature loss by avoiding feature flattening and to achieve depression score prediction. Tests on the AVEC2013 and AVEC2014 datasets reveal that our approach yields results on par with state-of-the-art techniques for detecting depression using video analysis. Additionally, our method has significantly lower computational complexity than mainstream methods.

## 1. Introduction

Depression is a mental illness, also known as Major Depressive Disorder (MDD), characterized by persistent and severe negative emotions. Around 350 million individuals globally are affected by MDD, according to data from the World Health Organization (WHO). Intense depression can trigger self-harm and suicidal tendencies in individuals [1,2]. Fortunately, severe depression can be effectively treated through appropriate psychological counseling, cognitive behavioral therapy, and medication [3]. Therefore, it is important to diagnose depression accurately for timely treatment and relief.

In the clinical diagnosis of mental health issues, professionals utilize self-report questionnaires, such as the Patient Health Questionnaire-9 (PHQ-9) [4] and the Beck Depression Inventory-II (BDI-II) [5] to assess the severity of depressive symptoms and behaviors. Additionally, they conduct interview assessments, including the Hamilton Depression (HAMD) Rating Scale [6]. However, these diagnostic methods are highly subjective, and their accuracy largely depends on the cooperation of individuals with depression and the expertise of mental health professionals. In such cases, clinicians may occasionally overdiagnose or misdiagnose depression.

Furthermore, clinical interventions are typically labor-intensive, expensive, and require a significant amount of specialized expertise in managing depressive states. In order to provide timely treatment for patients with depression, finding accurate and efficient depression detection technology is urgent [7,8].

Due to the fast evolution of computer vision and deep learning technologies, automatic depression analysis has become a research hotspot. Researchers focus on using visual nonverbal cues, such as facial expressions and movements, to identify and analyze depressive symptoms [9,10,11,12]. These methods are valuable because psychological studies show that individuals with depression can be identified through a series of objective visual cues [13,14,15]. These cues are automatically detected and analyzed, providing a new approach for early identification and intervention.

However, effectively encoding temporal patterns of facial features remains a technical challenge. To address this issue, researchers employ various deep learning techniques, including 3D-CNN [16], Long Short-Term Memory (LSTM) [17,18], and Convolutional Neural Networks (CNNs) [19], aiming to make breakthroughs in the field of depression recognition. Particularly noteworthy is the dual-stream RNN+3D-CNN model [10] proposed by the author Jazaery, marking the first application of fusion models in depression video detection. Jazaery’s model combines the strengths of RNNs and 3D-CNN, enabling the automatic learning of spatiotemporal features from continuous facial expressions, thereby enhancing the accuracy of depression detection. Although these deep learning methods make significant advancements in performance, their computational costs remain high, limiting their widespread use in real-time clinical applications. Consequently, future research needs to focus on reducing computational complexity further while maintaining high precision to facilitate broader real-time clinical application.

Past research indicates the crucial importance of temporal context between adjacent frames in video object detection [20,21]. Utilizing temporal context information in videos to aggregate and enhance input data is an effective strategy. Furthermore, temporal differencing techniques are widely used for capturing motion features [11,22]. By combining temporal difference features with original video features, motion features appearing in the video are effectively encoded. Inspired by the above points, this work proposes a lightweight model called Time-Context Enhanced Depression Detection Network (TCEDN). In the first stage, the model uses an attention mechanism to aggregate video frame features, aiming to enhance feature representation while reducing computational complexity. In the second phase, the model employs a learnable weight-enhanced temporal differencing technique to encode facial motion features related to depression. Subsequently, in order to address the issue of potentially losing spatial feature information when flattening CNN outputs to serve as inputs for Recurrent Neural Network (RNN) in the traditional CNN+RNN model, ConvLSTM [23] is employed in the third stage to further process the spatiotemporal information captured by the 3D convolutional network. Finally, the model utilizes a combination of 2-Dimensional Convolutional Neural Network (2D-CNN) and linear fully connected (FC) layers to obtain depression scores. Extensive experiments conducted on the public AVEC2013 [24] and AVEC2014 [25] datasets demonstrate the superiority of the proposed TCEDN in automatic video-based depression detection tasks, and it can be trained end-to-end.

The main contributions of this work are summarized as follows:

- We define an Attention-Weighted Aggregation Module (AWAM) to aggregate frame-level features of video representation, which reduces the model’s input while enhancing model performance, effectively lowering the computational complexity of the model.
- We propose a facial feature weighting module with self-learning weights, which integrates video raw features and facial movement features in a self-learning weight manner, effectively improving the accuracy of depression detection.
- We adopt a 3D-CNN optimized based on ConvLSTM to model the task of depression recognition, effectively reducing the potential loss of spatial feature information in the model concatenation process by avoiding feature flattening.
- We conduct end-to-end experiments on the corresponding datasets, showing that the TCEDN model performs comparably or even better than state-of-the-art methods. Furthermore, our approach demonstrates lower computational complexity while avoiding pre-training costs.

The structure of this work is outlined as follows: Section 2 delves into the related work, Section 3 presents the modules and methodologies of the model, Section 4 introduces the dataset along with its processing methods and the model evaluation metrics, Section 5 presents the experimental results and analysis, and, finally, Section 6 draws conclusions from the study.

## 2. Related Work

In this section, we provide an overview of video-based depression detection, discussing the exploration of depression prediction under different feature extraction methods. Based on clinical research, the facial cues associated with depression include reduced smiling, frowning, and a lack of eye contact [8,13,14,15]. Compared to healthy control groups, individuals with depression exhibit decreased head movements [26]. These cues support automatic depression detection. Traditional machine learning methods utilize handcrafted features like Local Phase Quantization (LPQ) [24] and Local Gabor Binary Patterns from Three Orthogonal Planes (LGBP-TOP) [25]. In contrast, deep learning techniques, such as CNN [19] and LSTM [17,18], automatically extract advanced features for depression prediction. Subsequently, we discussed feature enhancement based on temporal difference operations, as it is relevant to our research.

### 2.1. Hand-Crafted Methods

Early studies in the Audio/Visual Emotion Challenge (AVEC) initially emphasize handcrafted feature extraction for automated visual depression detection. Common facial descriptors used to gauge depression severity include LPQ [24], Local Phase Quantization from Three Orthogonal Planes (LPQ-TOP) [27], Local Binary Patterns from Three Orthogonal Planes (LBP-TOP) [28], LGBP-TOP [25], and Motion History Histogram (MHH) [29]. These features are then utilized in conjunction with machine learning techniques like decision trees, logistic regression, and Support Vector Regression (SVR) for depression prediction. Nevertheless, manual feature extraction methods often provide elementary feature representations, leading to limited efficacy in depression detection.

### 2.2. Deep Learning Methods

Deep learning techniques, especially 2D-CNN, 3D-CNN, and Transformer models, have been widely applied in the field of automatic depression detection based on videos due to their outstanding feature extraction capabilities [9,10,11,12].

2D-CNN analyzes image features in videos, such as facial expressions and body language, to identify individual depression symptoms. For example, Zhu et al. [30] proposed a deep learning architecture that utilizes two streams to process facial images and optical flow inputs, while Jan et al. [31] explored appearance information and encoded feature changes through 2D-CNN combined with a Feature Dynamic History Histogram (FDHH).

3D-CNN applies convolution operations in the third dimension (time) to process spatiotemporal information simultaneously, effectively capturing spatiotemporal features in videos, suitable for video classification and action recognition tasks. Jazaery et al. [31] captured spatiotemporal features at different scales using two Convolutional 3D (C3D) networks, while Melo et al. [32] extracted spatiotemporal features from different facial regions using two C3D networks and proposed a novel multiple instance learning method for depression detection by combining LSTM and multiple instance learning pooling.

The Transformer model, with its self-attention mechanism, has achieved great success in the field of natural language processing [33,34,35,36,37] and has shown potential in video analysis. Models based on Transformer, such as the Dual Attention and Element Recalibration Network (DAERNet) [38] and the Deep Local Global Attention CNN (DLGA-CNN) [33], enhance the understanding and analysis of complex scenes in depression video data through innovative attention allocation strategies.

Furthermore, ConvLSTM [23] combines the strengths of CNN and RNN, making it particularly suitable for processing video and image sequence tasks [39]. By integrating the spatial feature extraction capabilities of CNN with the temporal analysis capabilities of RNN, it significantly enhances the prediction accuracy of video analysis tasks. In our current work, we aim to introduce the ConvLSTM model into the field of depression video recognition, providing a new tool for the identification of depression.

### 2.3. Time-Difference Operation

The purpose of temporal differencing operations is to model changes in motion within videos. Currently, two types of temporal differencing operations, namely image differencing [40,41] and feature differencing [42,43,44], have been widely used for motion extraction. Image differencing is aimed at modeling motion in images and providing optical flow as a potential alternative representation of motion [40,41]. Network designs based on differencing operations utilize feature differencing. Various differential approaches have been proven to be effective methods for image motion modeling.

## 3. Proposed Method

**Figure 1:**
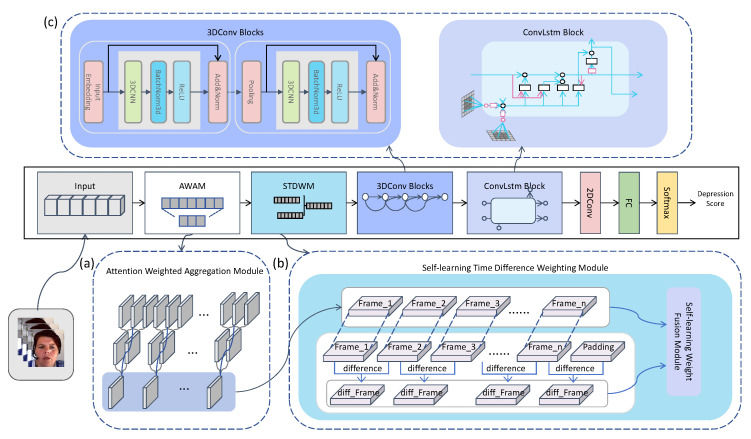
The proposed TCEDN framework, as shown in the figure, consists of three key components: (**a**) the Attention-Weighted Aggregation Module (AWAM) for video frames, (**b**) the Self-Learning Time-Difference Weighting Module (STDWM), (**c**) the Concatenated Network based on ConvLSTM and 3D-CNN. Video data are input into the model in the form of a series of frames, first passing through the Attention-Weighted Aggregation Module to highlight important information in the video. The aggregation module aggregates the weighted frames to generate a comprehensive feature representation. The aggregated features then enter the STDWM, which can intelligently fuse and enhance time-difference features, capturing subtle changes in facial expressions in the video. The fused features are further input into the ConvLSTM-based 3D-CNN network, processed, and finally mapped to a specific score using a traditional 2D-CNN regression network.

We have proposed a lightweight model named TCEDN specifically for automated depression detection based on video data. This model achieves this goal by aggregating video features and encoding facial motion features. The framework of the model consists of three key components, as depicted in Figure 1. Firstly, we designed the Attention-Weighted Aggregation Module (AWAM), which takes raw video data as the input and aggregates these data through an attention mechanism to generate aggregated and enhanced video frame encodings. Secondly, the Self-Learning Time-Difference Weighting Module (STDWM) processes the aggregated enhanced video features outputted by AWAM. In this module, we use self-learning weights to intelligently fuse the video frames with the time-difference features extracted from these frames, thereby integrating original video features and facial motion features. Finally, we utilized a 3D-CNN network optimized with ConvLSTM to encode the spatiotemporal features of the fused features output by the STDWM module, and mapped them to specific scores using a traditional 2D-CNN regression network. In the following sections, we will detail the design principles, the implementation methods of these three components, and how they collaborate to enhance the accuracy and efficiency of depression video detection.

### 3.1. Attention-Weighted Aggregation Module

**Figure 2:**
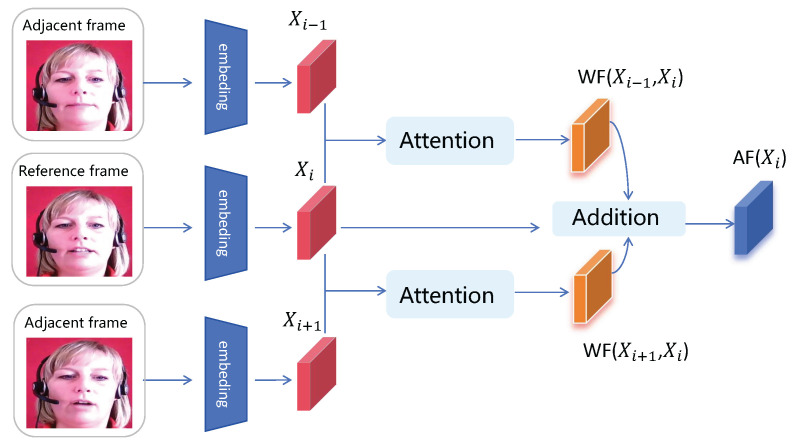
Attention-Weighted Aggregation Module. Feature aggregation module based on attention mechanism weighting, taking three adjacent frames as an example.

As shown in Figure 1a, we have designed an attention-weighted aggregation module. After passing the original video through the AWAM, we obtain the aggregated representation of video features. Taking the aggregation of adjacent three frames as an example, the specific implementation of this model is illustrated in Figure 2. Our goal is to use attention weights to indicate the importance of all adjacent frames to the reference frame for enhancement. Therefore, we incorporate an attention module into the model that computes cross-attention weights between the reference frame and adjacent frames to allocate pixel-wise aggregation weights at each frame. Specifically, we use the similarity weights computed between adjacent frames to perform element-wise multiplication with the reference frame, and then sum the weighted frames with the reference frame to obtain the final aggregated frame result. The specific formula is as follows:

(1) $$WF\left(X_{j},X_{i}\right)=\mathrm{Norm}\left(\frac{\phi \left(X_{j}\right)⊙\phi \left(X_{i}\right)}{\sqrt{d}}\right)⊙X_{i},$$

where $X_{i}$ is the reference frame, ϕ is a mapping function that transforms frame features into a space suitable for calculating attention scores, and d is a scaling factor used to stabilize the attention operation. To adapt to operations in the image domain, we use the normal function instead of the softmax function to ensure that the encoding weights can be element-wise multiplied with the target frame. $WF(X_{j},X_{i})$ represents “WeightFrame”, which is the result of attention weighting between the vectors of two adjacent frames. On this basis, we aggregate adjacent frames:

(2) $$AF\left(X_{i}\right)=\alpha \mathrm{WF}\left(X_{i-1},X_{i}\right)+\gamma X_{i}+\beta \mathrm{WF}\left(X_{i+1},X_{i}\right),$$

where $AF(X_{i})$ represents “AggregationFrame”, which is the result of the three-frame aggregation for the reference frame $X_{i}$. In particular, α, β and γ are hyperparameters used during frame addition. We typically set the sum of the three parameters to 1. We usually use values between 0.05 and 0.1 for the first two hyperparameters to avoid introducing too much noise during the aggregation process.

### 3.2. Self-Learning Time-Difference Weighting Module

**Figure 3:**
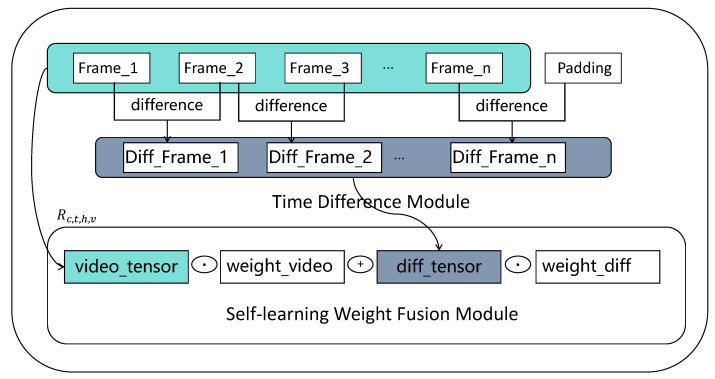
Self-Learning Time-Difference Weighting Module. The proposed STDWM, as shown in the figure, consists of two components: the Time-Difference Module and the Self-Learning Weight Fusion Module (SWFM). The raw video data are first processed by the Time-Difference Module to obtain facial motion features, which are then combined with the primitive facial features and weighted through the SWFM to obtain enhanced video features.

As shown in Figure 1b, this study proposes a novel approach to generate robust representations of facial changes by encoding both the raw facial features and facial motion features. The specific implementation of this model is illustrated in Figure 3. We introduce temporal difference blocks to simulate facial motion changes and design a Self-Learning Weight Fusion Module (SWFM) to enhance the fusion of raw video features with facial change features. The temporal difference blocks explore short-term facial changes by computing low-order differences, while the SWFM weights and fuses features using a learnable weight matrix to form an enhanced representation of facial motion features.

#### 3.2.1. Time-Difference Module

To generate a robust representation of facial changes, it is crucial to encode the original facial features and facial motion features. These variations can help the model analyze video segments with similar facial expression changes. With this motivation, we first propose a structure called temporal difference block to model facial motion changes. Let $V\in R^{B\times W\times H\times C\times T}$ be defined as the input aggregated video features, where B, W, H, C, and T represent the batch size, feature width, feature height, number of channels, and temporal depth (number of frames). As shown in Figure 3, the temporal difference operation is defined as:

(3) $$D_{t}=\left|V_{t}-V_{t-s}\right|,$$

where $D_{t}$ is the output of differential operation with the temporal depth t, and represents s-th order difference. In our implementation, we maintain the depth of the output to be equal to the input feature map, and we add zeros to the input features when performing the operation. Since differences with too large strides represent a large distance between two frames of data, which leads to a lot of noise and fewer features in the facial change features, the difference block is designed to explore short changes, and therefore low-order differences are often used, such as 1, 2, and 3.

#### 3.2.2. Self-Learning Weight Fusion Module

Based on the Time-Difference Module, we design a Self-Learning Weight Fusion Module (SWFM) to enhance the fusion of original video features and facial change features. As shown in Figure 3, SWFM utilizes two learnable weight matrices of the same size as the video features to weight the original video feature block and the facial motion feature block. The specific operation involves taking the dot product of the temporal difference features and the original features using the learnable weight matrices, and then adding the results. This operation is defined as:

(4) $$R_{w,h,c,t}=\left(V_{w,h,c,t}⊙W_{v}\right)+\left(D_{w,h,c,t}⊙W_{d}\right),$$

where $R_{w,h,c,t}$ represents the result of the operation, $V_{w,h,c,t}$ denotes the original video features, and $D_{w,h,c,t}$ signifies the facial motion features. $W_{v}$ and $W_{d}$ are the self-learning weight matrices corresponding to different features. We combine the two types of weighted features to form an enhanced depressive video stream feature based on facial motion characteristics.

### 3.3. Depression Video Recognition Network

In this section, we propose an optimized 3D-CNN network based on ConvLSTM. The 3D-CNN in our model adopts a residual structure, enhancing the network’s depth and feature encoding capability through residual blocks and skip connections. Additionally, to preserve spatial features, we introduce ConvLSTM to handle spatiotemporal feature tensors and map features to specific scores through a 2D-CNN regression network.

#### 3.3.1. 3D-CNN Block

The 3D-CNN is a deep learning architecture that extends traditional 2D-CNN into three-dimensional space, allowing it to process volumetric data such as video frame sequences, three-dimensional medical images, or any other form of three-dimensional datasets. Three-Dimensional CNN can capture spatial features (like shape and texture) and temporal features (like motion) simultaneously, making them perform exceptionally in areas such as video processing and action recognition.

We propose a 3D-CNN block (Figure 1c), which is composed of 3D convolutional layers, Batch Normalization (BN), the ReLU activation function, and a down-sampling layer modified based on 3D convolution. Building upon this, we follow the structure of the ResNet network, treating each 3D-CNN block as a residual block, where the 3D-CNN block is the fundamental unit for constructing the network. This design allows the network to learn the residual function from input to output and alleviates the vanishing gradient problem through skip connections, enabling the network to be trained deeper and achieve better feature encoding capabilities.

Overall, by progressively extracting and abstracting features, the 3D-CNN model network is able to learn complex patterns in the data, which are crucial for performing tasks, such as video classification and action recognition. In our model, the 3D-CNN model significantly optimizes the performance of the model. We think that the use of a residual 3D-CNN model can provide a level of global feature optimization from both temporal and spatial perspectives.

#### 3.3.2. ConvLSTM Module

**Figure 4:**
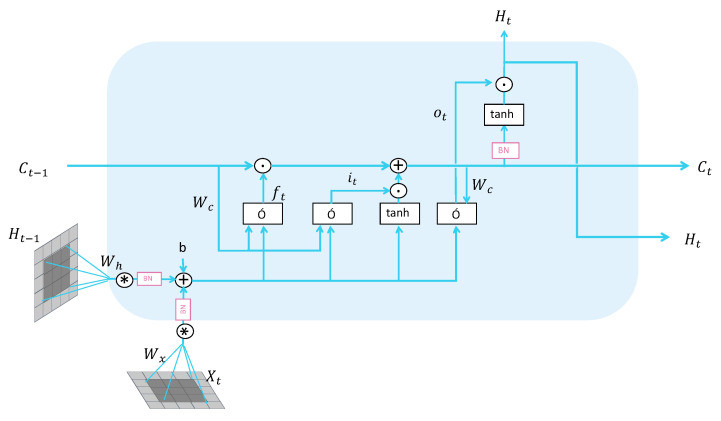
ConvLSTM is a hybrid model that combines CNN and LSTM. In the figure, the arrows indicate the direction of data flow, ∗ denotes the convolution operation, σ represents the sigmoid activation function. By integrating the spatial feature extraction capability of CNN and the temporal modeling capability of LSTM, it can effectively handle spatiotemporal data and achieve good performance in tasks such as video prediction and time-series forecasting.

In the traditional domain of depression, the method to optimize 3D-CNN on temporal features involves cascading with LSTM. However, a major drawback of this approach in handling spatiotemporal data is that it disrupts the spatial features encoded by 3D-CNN at the junctions. Constrained by the input requirements of the LSTM model itself, two-dimensional facial features are stretched into one-dimensional tensors, thus compromising the spatial features. To overcome this problem, we use ConvLSTM to process the spatiotemporal feature tensors encoded by 3D-CNN. ConvLSTM applies convolution operations at each time step, aiding in capturing spatial information within the temporal data. As illustrated in Figure 4, the operation of ConvLSTM used for single-step prediction is defined as follows:

(5) $$i_{t}=\sigma \left(W_{xi}*X_{t}+W_{hi}*H_{t-1}+W_{ci}\circ C_{t-1}+b_{i}\right),$$

where ∗ denotes the convolution operation, and ∘ represents the Hadamard product, σ represents the sigmoid activation function. $i_{t}$ is the output of the input gate. $X_{t}$ is the input at the current time step. $H_{t-1}$ is the hidden state at the previous time step. $C_{t-1}$ is the cell state from the previous time step. $W_{xi}$, $W_{hi}$, and $W_{ci}$ are the weight parameters of the input gate. $b_{i}$ is the bias term of the input gate.

(6) $$f_{t}=\sigma \left(W_{xf}*X_{t}+W_{hf}*H_{t-1}+W_{cf}\circ C_{t-1}+b_{f}\right),$$

where $f_{t}$ is the output of the forget gate. $X_{t}$ is the input at the current time step. $H_{t-1}$ is the hidden state at the previous time step. $C_{t-1}$ is the cell state from the previous time step. $W_{xf}$, $W_{hf}$, and $W_{cf}$ are the weight parameters of the forget gate. $b_{f}$ is the bias term of the forget gate.

(7) $$C_{t}=f_{t}\circ C_{t-1}+i_{t}\circ \tanh\left(W_{xc}*X_{t}+W_{hc}*H_{t-1}+b_{c}\right),$$

where tanh represents the hyperbolic tangent activation function. The cell state is generated based on the output of the input gate, the cell state from the previous time step, and other input parameters.

(8) $$o_{t}=\sigma \left(W_{xo}*X_{t}+W_{ho}*H_{t-1}+W_{co}\circ C_{t}+b_{o}\right),$$

where $o_{t}$ is the output of the output gate. $X_{t}$ is the input at the current time step. $H_{t-1}$ is the hidden state at the previous time step. $C_{t-1}$ is the cell state from the previous time step. $C_{t}$ represents the memory cell at the current time step. $W_{xo}$, $W_{ho}$, and $W_{co}$ are the weight parameters of the output gate. $b_{o}$ is the bias term of the output gate.

(9) $$H_{t}=o_{t}\circ \tanh\left(C_{t}\right),$$

where $H_{t}$ is the hidden state at the current time step. The hidden state is updated based on the output gate and the current cell state. ConvLSTM’s multi-step prediction is similar to single-step prediction; simply take the output of the single-step prediction as the input for the next time step and perform recursive calculations.

On this basis, we use a traditional 2D-CNN regression network to map the final three-dimensional features to specific scores.

## 4. Experimental Dataset and Settings

In this section, we first introduce the datasets used for training and testing the model, as well as the steps involved in preprocessing the data in the datasets. Then, we provide a detailed explanation of the model’s implementation details, including various types of model parameters and the metrics used to evaluate the model’s performance.

### 4.1. Datasets and Pre-Processing

- **Experimental Setup:** TCEDN’s performance is being assessed through depression detection experiments using video data from the AVEC2013 and AVEC2014 public datasets. BDI-II depression scores are being used as labels for these experiments.
- **AVEC2013 Dataset:** the AVEC2013 dataset consists of 150 videos divided into training, validation (development), and test sets, each containing 50 videos with corresponding depression score labels.
- **AVEC2014 Dataset:** AVEC2014 involves two tasks during video recording: “Freeform” and “NorthWind”. Each task includes training, validation, and test sets, with each set comprising 50 videos with depression scores.
- **BDI-II:** The AVEC2013 and AVEC2014 datasets use BDI-II scores as labels for video predictions. The severity of depression can be categorized into four levels: minimal (0–13), mild (14–19), moderate (20–28), and severe (29–63).
- **Data Processing:** Uniformly sample 48 or 66 frames for each given video. For each frame, we employed the Multi-Task Cascaded Convolutional Networks (MTCNN) [45] for face detection and alignment, followed by cropping the portion containing the eyes (sized at 270 × 80) to form the video data of the eye region. Subsequently, each video sample of the eye region was used as input for the TCEDN model.

### 4.2. Implement Details and Evaluation Metrics

We implement the TCEDN method using the PyTorch framework and deploy the model for training on an RTX 4090 GPU from NVIDIA, based in the United States. The model is trained end-to-end on the original video data to avoid additional pre-training costs. We utilize Mean Squared Error (MSE) as the loss function, defined as:

(10) $$\mathrm{MSE}=\frac{1}{n}\sum_{i=1}^{n}{\left(y_{i}-{\hat{y}}_{i}\right)}^{2},$$

similar to existing methods, we use Mean Absolute Error (MAE) and Root Mean Square Error (RMSE) as metrics to evaluate model performance, defined as:

(11) $$\mathrm{MAE}=\frac{1}{n}\sum_{i=1}^{n}\left|y_{i}-{\hat{y}}_{i}\right|,$$

(12) $$\mathrm{RMSE}=\sqrt{\frac{1}{n}\sum_{i=1}^{n}{\left(y_{i}-{\hat{y}}_{i}\right)}^{2}}.$$

**Figure 5:**
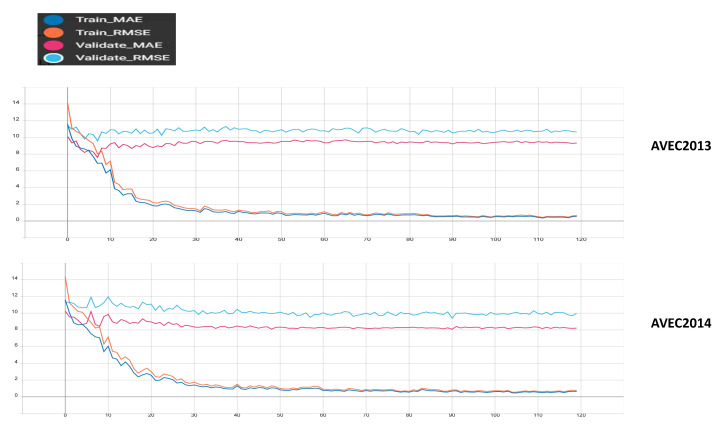
The process of training models on AVEC2014 and AVEC2013 datasets. We recorded the MAE and RMSE metrics for both the training and validation sets to evaluate the model’s performance. As training progressed, we observed a gradual decrease in the MAE and RMSE metrics, eventually leveling off.

The model is optimized using the ADAM algorithm with a learning rate of 0.000025. The model decreases the learning rate every 30 runs, with a decay factor set to 0.8. The maximum training epochs for this model are set to 120, with a batch size of 4. Specific training details are illustrated in Figure 5. As training progresses, we observe a synchronized decrease in the metrics of the training set and the validation set. Starting from the tenth epoch, they begin to decrease at different rates, and around the 50th epoch, the metrics of both the training set and the validation set tend to plateau.

## 5. Results and Analysis

The section is organized as follows: in the first part, we analyze the impact of different time steps on model performance. In the second part, we examine the experiments and performance of each module to determine the necessity of each module in the model. In the third part, we compare the effects of data from different regions on model performance to elucidate why we conducted experiments using the eye region. In the fourth part, we compare the performance of our model with mainstream models to demonstrate the advantages of our model. In the fifth part, we analyze the test set video samples and discuss the strengths and limitations of the model in practical applications. In the sixth part, we conducted tests on the robustness of the model to simulate its application in different clinical environments. In the final part, we showcase the distribution of weights in the model through visual analysis to help readers better understand the characteristics and rationality of the model.

### 5.1. Influence of Different Time Steps on TCEDN

Different temporal difference step sizes may affect the performance of the TCEDN. To minimize the impact of other data augmentation modules on the ablation results, we use the temporal difference module as the sole data augmentation module. We conducted experiments on the corresponding datasets, as shown in Table 1, the model achieved the best performance in MAE and RMSE when difference steps were set to 1. However, as the difference steps increased to 2, 3, and 4, the performance of the model in terms of MAE and RMSE both declined. We speculate that increasing the step size leads to the time-difference calculation involving frames that are further apart, making it more difficult for the time-difference results to accurately capture the characteristics of facial expression changes while also potentially introducing more noise. It is worth noting that the decrease in RMSE is relatively small, which may be due to the lower sensitivity of RMSE to minor noise. Therefore, choosing an appropriate difference step is crucial to ensuring the accuracy and stability of the TCEDN model.

In particular, we have discovered that features fused from time differences of various step lengths can also yield good results, although this does indeed introduce an additional computational burden.

### 5.2. Evaluation of Different Modules in TCEDN

As mentioned earlier, our TCEDN architecture consists of three key components aimed at improving the performance of depression recognition tasks. Firstly, we utilize an attention-weighted mechanism to aggregate contextual information, which not only reduces computational load but also enhances the representational capacity of features. Secondly, the Self-Learning Time-Difference Weighting Module (STDWM) significantly enhances the representational capacity of facial video and motion features by fusing them. Lastly, we employ a hybrid model of 3D-CNN and ConvLSTM to further enhance the modeling capability of spatiotemporal feature sequences. To comprehensively evaluate the role of each component in the TCEDN architecture, we conducted a series of ablation studies and presented the results in Table 2. By gradually integrating each component, we assessed their impact on overall performance to determine their necessity. Furthermore, we compared traditional cascade-based data aggregation methods and attention-based data aggregation algorithms, as well as the performance of traditional LSTM and ConvLSTM in spatiotemporal feature modeling. Through these comparative experiments, we revealed the advantages and disadvantages of different methods, providing a basis for further model optimization.

Table 2 presents a comparison between the traditional 3D-CNN+LSTM model and our proposed TCEDN architecture. The data show that the 3D-CNN+LSTM configuration performs poorly on MAE and RMSE metrics, while the 3D-CNN+ConvLSTM configuration is significantly better than the former. This highlights the importance of combining the spatiotemporal feature extraction capability of 3D-CNN with the sequence processing capability of ConvLSTM to enhance task performance, especially the advantage of ConvLSTM in preserving spatial features.

Furthermore, after the introduction of the Self-Learning Time-Difference Weighting Module (STDWM), the model’s average MAE and RMSE improved by 0.2 to 0.7, confirming the effectiveness of STDWM in enhancing feature encoding. We speculate that this is due to the effective weighting of facial video features and facial motion features at a fine granularity by the SWFM, thereby facilitating better feature fusion.

At the same time, we also found that the AWAM significantly improved the accuracy of depression recognition, while the traditional feature aggregation module based on adjacent frame splicing (SpliceConv) may have a negative effect. By comparing the experimental group containing AWAM and STDWM, we found that these two modules can have a positive impact on depression recognition at the same time, and their effects can be superimposed.

Ultimately, the complete TCEDN model demonstrated outstanding performance on the AVEC2013 and AVEC2014 datasets, achieving significant improvements in performance. This can be attributed to the synergistic effects and effective integration of various modules: the AWAM module highlighted key domains relevant to depression recognition frameworks, the STDWM module enhanced the capture of critical facial motion features through learnable weights, 3D-CNN provided rich spatiotemporal information, and ConvLSTM bolstered the model’s ability to handle time-series data. This multi-module fusion strategy not only enhanced the model’s predictive accuracy but also strengthened its capability to recognize complex emotional states.

### 5.3. Slicing Effects in Different Regions

Different regions of video features may affect the performance of TCEDN. Table 3 displays the performance of the TCEPN network using complete facial slices, slices around the eyes, and a combination of both on the corresponding datasets. On the AVEC2013 dataset, the TCEDN network using eye slice data outperformed the TCEDN network using facial slice data, and the same situation occurred on the AVEC2014 dataset. These results suggest that there is a certain degree of redundancy in the spatiotemporal information of the global facial region. They also indicate that the spatial structure and temporal correlation of the local eye region are more important for predicting depression using the TCEDN network. Specifically, later in the text, we visualize the weight distribution of the depression recognition model, and the visualization results are consistent with those in Table 3.

### 5.4. Comparison with State-of-the-Art

As shown in Table 4, we present the current state-of-the-art methods, primarily covering manual feature recognition and deep learning recognition. The specific comparative results are as follows.

#### 5.4.1. Performance Comparison of Methods Based on the AVEC2013 Dataset

Table 5 presents the performance comparison of our TCEDN method with the current state-of-the-art methods on the development (test) set of the AVEC2013 dataset. To assess the effectiveness of the models, we ensured fairness by selecting models with similar training weights for comparison. We utilized MAE and RMSE as evaluation metrics, quantifying computational complexity based on the number of network parameters (Param.) (M) and the number of floating-point operations per second (FLOPs) (G). According to the results in Table 5, our TCEDN method achieved performance in terms of RMAE comparable to other recent methods. Specifically, TCEDN’s RMAE was 7.83, surpassing all current mainstream handcrafted feature recognition methods. Our model exhibited the best or highly competitive performance in terms of RMSE and MSE. Particularly, the MSN model achieved the second-lowest RMSE through its efficient deep 3D-CNN architecture and a significant number of computational parameters. Our TCEDN method optimized features using ConvLSTM and reduced the number of processed features effectively by incorporating attention aggregation modules, thereby effectively reducing computational complexity.

#### 5.4.2. Performance Comparison of Methods Based on the AVEC2014 Dataset

Table 6 illustrates the performance comparison of our TCEDN method with the current state-of-the-art methods on the development (test) set of the AVEC2014 dataset. Similar to the model’s performance on the AVEC2013 dataset, our TCEDN achieved competitive performance. However, the MAE produced by our TCEDN was slightly lower than models like MSN and DAERNet. We attribute this difference to the fact that models like MSN and DAERNet require pretraining on large-scale image datasets [53], thus having more prior knowledge, while TCEDN is designed as a lightweight end-to-end deep learning model with lower computational complexity and performance.

#### 5.4.3. Comparison of Computational Complexity of Different Methods

Following in [11,44,55], two representative indicators such as the Param.(M) and FLOPs(G) are adopted to measure the computational complexity. Table 7 presents a comparison of computational complexity for seven typical CNN networks, including ResNet-50, MDN-50, TS-RNN+3D-CNN (two-stream RNN+3DCNN), MSN, MTDAN, OpticalDR, and our TCEDN. From Table 7, it can be seen that among the seven CNN networks used, our TCEDN achieves competitive computational complexity on both the corresponding datasets, with a parameter count of 5.08 M and FLOPs of 2.85 G. Under these conditions, TCEDN still provides performance comparable to other methods. Especially on the AVEC2013 dataset, our TCEDN achieved the best RMAE of 7.83, while on the AVEC2014 dataset, it delivered good RMSE and MAE results. Despite MTDAN having the lowest computational complexity, our model outperformed MTDAN in accuracy on both datasets, demonstrating the performance advantage of our TCEDN as a lightweight deep learning model.

In this study, we utilized the AWAM module to compress and enhance video data to improve the efficiency of the model. Specifically, we compressed the video data in the AVEC2013 dataset to 64 frames and in the AVEC2014 dataset to 16 frames. This was carried out to reduce the computational complexity during model runtime. To further illustrate the advantages of our approach, we compared it with models proposed by Melo et al. [32] and Jazaery et al. [10]. These models have varying input frame ranges from 60 frames to 360 frames, indicating that our model is more streamlined in terms of input data volume.

Additionally, we contrasted our model with the MDN-50 and MSN models. While these models also employed methods of sampling a small number of frames, they utilized 3D-CNNs with 50 layers and 69 layers, respectively, making their model structures more complex with a larger number of parameters. In comparison, our model achieved excellent results by optimizing with the addition of the covlstm model on top of the 3D-CNN model, using only 16 layers of 3D-CNN. This approach reduces the number of model computations and significantly reduces the number of parameters, thereby decreasing the computational complexity of the model.

Overall, through the compression enhancement of the AWAM module and the optimization with the CovLSTM model, our model maintains outstanding performance while reducing computational complexity.

### 5.5. Error Analysis of TCEDN

**Figure 6:**
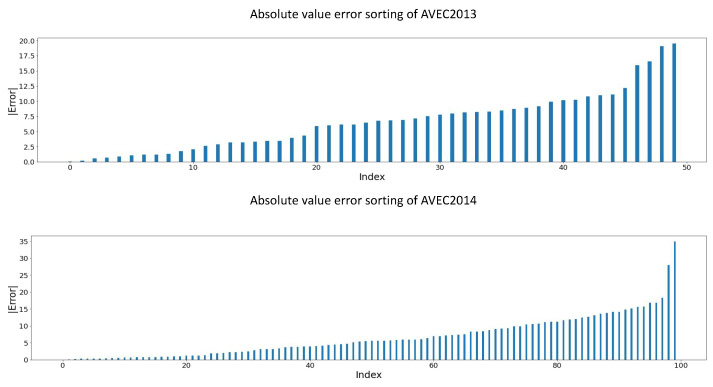
The error distribution of videos with the minimum absolute error to videos with the maximum absolute error on two public datasets.

**Figure 7:**
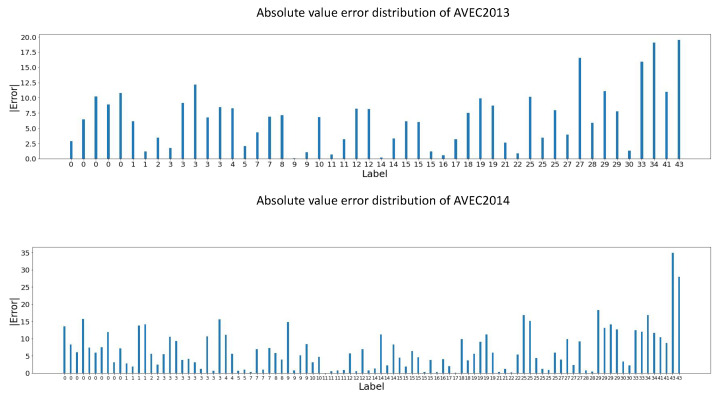
Absolute error distribution of videos from lowest to highest depression scores on two public datasets.

To deepen our understanding of the model’s performance in clinical settings and provide more detailed model information, we conducted error analysis on each test video sample from the AVEC2013 and AVEC2014 datasets. In Figure 6 and Figure 7, we respectively present the absolute error distributions of the two datasets: Figure 6 illustrates the absolute error distribution of videos from the lowest to the highest depression scores, while Figure 7 shows the error distribution from the video with minimized absolute error to the one with maximized absolute error. These visualizations offer us an intuitive grasp of the model’s performance, aiding in further analysis.

As shown in Figure 6 and Figure 7, we observe that the absolute errors are roughly uniformly distributed between 0 and 12.5, with only a few outliers exceeding this range. Specifically, our model achieved errors below 6.0 in over 55% of the samples in the AVEC2013 and AVEC2014 datasets. This minimal level of error mainly reflects errors between adjacent categories and errors between category boundary scores (for example, predicting a score of 28, indicating moderate severity, while the actual score is 22, also indicating moderate severity). The most extreme cases occur when errors exceed 16, often leading to misclassifying individuals with mild depression as having severe depression or vice versa. On the AVEC2013 and AVEC2014 datasets, the most severe cases occurred in 3 and 5 video samples, respectively. As indicated by the analysis in conjunction with Figure 7, these extreme cases often involve patients with the highest depression scores. These results suggest that our model performs well in clinical applications with a low probability of severe misclassifications. However, the model performs poorly in diagnosing patients with the most severe depression. By statistically analyzing multiple samples with the highest depression scores, we found that the model consistently provides predicted values relatively smaller than the label values, indicating a tendency to offer conservative estimates of the depression severity for patients with severe depression.

### 5.6. Robustness Analysis of TCEDN

To further evaluate the robustness of the model, we subjected the input video data to various treatments. These processes include rotating the data at different angles, introducing various types of noise, and applying randomly sized masks. According to the results in Table 8, we observed that using different angles of rotation adversely affected the model’s performance. It is worth noting that the impact of different sizes of rotation angles on the model was generally consistent. Additionally, introducing different types of noise also had a negative impact on the model’s performance, with the effect of random noise being relatively minor. The addition of randomly sized masks to facial data had minimal impact on the model’s performance. Overall, while different treatments did influence the model, the degradation in model performance was not significant. The model exhibited strong robustness, which is beneficial for its application in various clinical scenarios.

### 5.7. Feature Visualization of TCEDN

**Figure 8:**
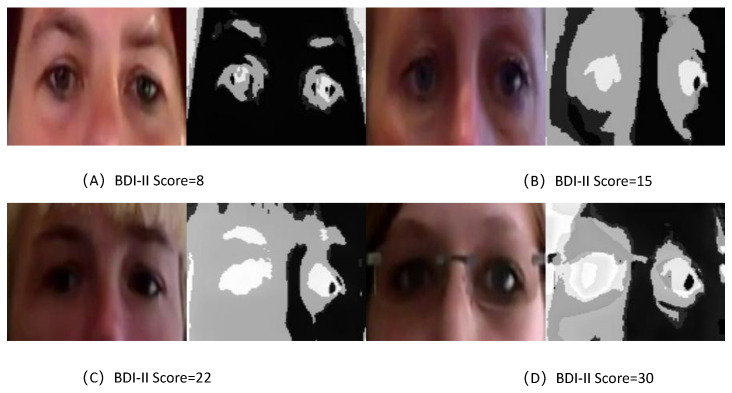
A visualization of the eye slice features of TCEDN on the AVEC2013 dataset: (**A**) Feature visualization for a BDI-II score of 8. (**B**) Feature visualization for a BDI-II score of 15. (**C**) Feature visualization for a BDI-II score of 22. (**D**) Feature visualization for a BDI-II score of 30.

**Figure 9:**
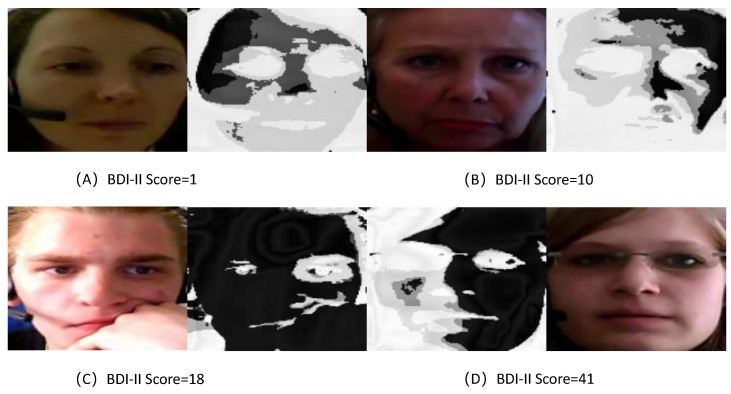
A visualization of the facial slice features of TCEDN on the AVEC2013 dataset: (**A**) Feature visualization for a BDI-II score of 1. (**B**) Feature visualization for a BDI-II score of 10. (**C**) Feature visualization for a BDI-II score of 18. (**D**) Feature visualization for a BDI-II score of 41.

**Figure 10:**
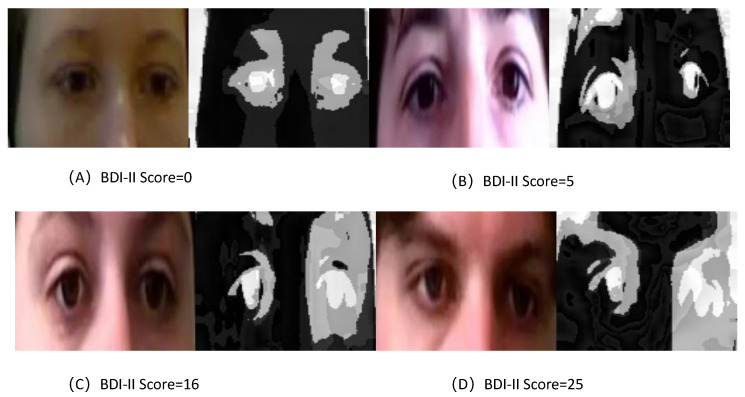
A visualization of the ocular slice features of TCEDN on the AVEC2014 dataset: (**A**) Feature visualization for a BDI-II score of 0. (**B**) Feature visualization for a BDI-II score of 5. (**C**) Feature visualization for a BDI-II score of 16. (**D**) Feature visualization for a BDI-II score of 25.

**Figure 11:**
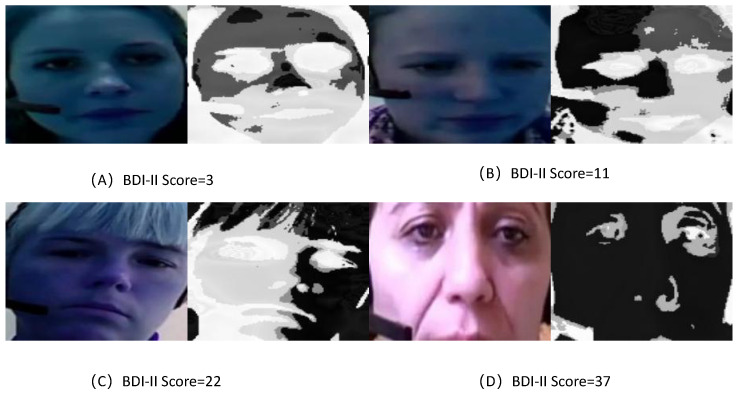
A visualization of the facial slice features of TCEDN on the AVEC2014 dataset: (**A**) Feature visualization for a BDI-II score of 3. (**B**) Feature visualization for a BDI-II score of 11. (**C**) Feature visualization for a BDI-II score of 22. (**D**) Feature visualization for a BDI-II score of 37.

To visually assess the learned feature maps of TCEDN, we employ the Gradient-Weighted Class Activation Mapping (Grad-CAM) [56] method to visualize how the feature weights in TCEDN differentiate facial images associated with depression. We extract weight maps from the ConvLSTM network and superimpose them onto the original facial images. Facial regions that aid in depression prediction are highlighted with lighter colors. We compare the visualization results of facial region slices and eye region slices on the corresponding datasets. As shown in Figure 8, Figure 9, Figure 10 and Figure 11, our TCEDN focuses on ocular activity; in almost all samples, the eye features are prominently displayed, with less emphasis on facial activity. We believe that the emphasis on ocular features by the TCEDN model gives it better performance on the eye slice dataset, which is consistent with our observations of its effectiveness. In addition, for the eye dataset, features in the eyebrow region and pupil region are more prominent. This is consistent with clinical research findings that individuals with depression may exhibit more negative facial expressions, such as furrowed brows and delayed eye contact, which may be related to their persistent low mood state.

## 6. Conclusions

This study proposes a lightweight Time-Context Enhanced Depression Detection Network (TCEDN) for automatic depression detection based on videos. The proposed TCEDN method consists of three key modules: an Attention-Weighted Aggregation Module (AWAM) for aggregating video features, a Self-Learning Time-Difference Weighting Module (STDWM) for modeling depressive facial motion behavior, and an Enhanced 3D-CNN network based on ConvLSTM for enhancing time-series prediction and spatial feature modeling. To evaluate the performance of the TCEDN method, two benchmark datasets, AVEC2013 and AVEC2014, are used for depression detection in facial videos. The experimental results demonstrate that the proposed TCEDN method achieves comparable or even better performance than state-of-the-art methods in video-based depression detection tasks at extremely low computational costs. Furthermore, we recognize the importance of handling the ethical issues related to depression detection with care. The use of depression detection models should be approached cautiously to minimize associated risks. This includes avoiding the disclosure of personal information of individuals with depression, preventing the misuse and misinterpretation of depression detection technology. In future works, we aim to introduce supplementary modalities like audio and text modalities in addition to the video modality, while ensuring the low computational complexity of the model to further enhance its performance.

## Figures and Tables

**Table 1:** Performance comparison of difference steps in STDWM.

Difference Steps	AVEC2013		AVEC2014	
	**RMSE**↓	**MAE**↓	**RMSE**↓	**MAE**↓
step_1	**8.03**	**6.61**	**7.91**	**6.48**
step_2	9.00	6.99	8.10	6.68
step_3	8.97	7.36	8.24	6.74
step_4	9.04	7.21	8.04	6.58
fusion	8.33	6.77	7.99	6.55

The magnitude of the step size represents the distance between the frames when calculating the time difference. In our table, we use bold text to indicate the best results. Additionally, in the table header, a downward arrow signifies that a smaller value indicates better performance.

**Table 2:** Analysis of the error rate of depression detection in each module of TCEDN.

Dataset	Methods	RMSE ↓	MAE ↓
AVEC2013	3D-CNN	9.39	7.68
	3D-CNN + Lstm(baseline)	9.28	7.37
	3D-CNN + ConvLstm	8.78	7.32
	STDWM + 3D-CNN+ConvLstm	8.03	6.61
	AWAM + 3D-CNN+ConvLstm	8.67	6.69
	SpliceConv + 3D-CNN + ConvLstm	8.51	6.98
	AWAM + STDWM + 3D-CNN + ConvLstm(ours)	**7.83**	**6.48**
AVEC2014	3D-CNN	8.90	7.43
	3D-CNN + Lstm(baseline)	9.20	7.22
	3D-CNN + ConvLstm	8.24	6.68
	STDWM + 3D-CNN + ConvLstm	7.91	6.48
	AWAM + 3D-CNN + ConvLstm	8.02	6.50
	SpliceConv + 3D-CNN + ConvLstm	9.00	7.12
	AWAM + STDWM + 3D-CNN + ConvLstm(ours)	**7.82**	**6.30**

In our table, we use bold text to indicate the best results. Additionally, in the table header, a downward arrow signifies that a smaller value indicates better performance.

**Table 3:** Performance comparison of slices from different regions.

Methods	AVEC2013		AVEC2014	
	**RMSE**↓	**MAE**↓	**RMSE**↓	**MAE**↓
eyes slice	**7.83**	**6.48**	**7.82**	**6.30**
face slice	8.78	7.32	9.57	7.12
fusion	8.93	6.91	8.82	7.11

In our table, we use bold text to indicate the best results. Additionally, in the table header, a downward arrow signifies that a smaller value indicates better performance.

**Table 4:** Depression video recognition methods.

Methods	Year	Method Category
Baseline [24]	2013	HM
MHH + PLS [29]	2013	HM
MHI + SVR [46]	2014	HM
LPQ + 1-NN [47]	2014	HM
LPQ-TOP + MFA [27]	2015	HM
rPPG + LGBP-TOP [48]	2023	HM
Two-stream CNNs [30]	2017	DLM
Two-stream 3DCNN [32]	2019	DLM
DepressNet [49]	2020	DLM
ResNet-50 [11]	2021	DLM
Two-stream RNN + 3DCNN [10]	2021	DLM
DLGA-CNN [33]	2021	DLM
MDN-50 [11]	2021	DLM
DAERNet [38]	2021	DLM
LQGDNet [50]	2021	DLM
MSN [51]	2022	DLM
Behavior primitives [12]	2022	DLM
MTDAN [22]	2023	DLM
OpticalDR [52]	2024	DLM

In this table, ‘HM’ stands for hand-crafted methods, and ‘DLM’ stands for deep learning methods.

**Table 5:** Performance comparison of methods based on the AVEC2013 dataset.

Methods	Year	RMSE ↓	MAE ↓	Param. ↓	FLOPS ↓
Baseline [24]	2013	13.61	10.88	–	–
MHH + PLS [29]	2013	11.19	9.14	–	–
LPQ + 1-NN [47]	2014	9.72	7.86	–	–
LPQ-TOP + MFA [27]	2015	10.27	8.22	–	–
Two-stream CNNs [30]	2017	9.82	7.58	–	–
Two-stream 3DCNN [32]	2019	8.26	6.40	–	–
DepressNet [49]	2020	8.19	6.30	–	–
ResNet-50 [11]	2021	8.81	6.92	63	12.22
DLGA-CNN [33]	2021	8.39	6.59	–	–
Two-stream RNN + 3DCNN [10]	2021	8.28	7.37	33.64	8.51
MDN-50 [11]	2021	8.13	6.39	21	7.40
DAERNet [38]	2021	8.13	6.28	–	–
LQGDNet [50]	2021	8.20	6.38	–	–
MSN [51]	2022	7.90	**5.98**	77.70	164.90
Behavior primitives [12]	2022	8.10	6.16	–	–
rPPG + LGBP-TOP [48]	2023	8.01	6.43	–	–
MTDAN [22]	2023	8.08	6.14	**0.90**	**1.97**
OpticalDR [52]	2024	8.48	7.53	67.28	4.36
Ours	2024	**7.83**	6.48	5.08	2.85

In our table, we use bold and underline to highlight the best and second-best results, and we use MAE and RMSE to measure the effectiveness of the model and quantify the computational complexity based on the number of network parameters (M) and the number of floating-point operations per second (G). In the table header, a downward arrow signifies that a smaller value indicates better performance.

**Table 6:** Performance comparison of methods based on the AVEC2014 dataset.

Methods	Year	RMSE ↓	MAE ↓	Param. ↓	FLOPS ↓
Baseline [25]	2014	10.86	8.86	–	–
LPQ + 1-NN [47]	2014	10.27	8.20	–	–
MHHPLS [29]	2014	10.50	8.44	–	–
MHI + SVR [46]	2014	9.84	8.46	–	–
Two-stream CNNs [30]	2017	9.55	7.47	–	–
CNN + DTL [54]	2017	9.43	7.74	–	–
VGG-Face + FDHH [31]	2017	8.04	6.68	–	–
Two-stream 3DCNN [32]	2019	8.31	6.59	33.64	8.51
DepressNet [49]	2020	8.55	6.39	–	–
Two-stream RNN + 3DCNN [10]	2021	9.22	7.20	–	–
ResNet-50 [11]	2021	8.40	6.79	63	12.22
DLGA-CNN [33]	2021	8.30	6.51	–	–
MDN-50 [11]	2021	8.16	6.45	21	7.4
Behavior primitives [12]	2022	8.30	6.78	–	–
DAERNet [38]	2022	8.07	6.14	–	–
MSN [51]	2022	**7.61**	**5.82**	77.70	164.90
MTDAN [22]	2023	7.93	6.35	**0.90**	**1.97**
rPPG + LGBP-TOP [48]	2023	8.49	6.57	–	–
OpticalDR [52]	2024	8.82	7.89	67.28	4.36
Ours	2024	7.82	6.30	5.08	2.85

In our table, we use bold and underline to highlight the best and second-best results, and we use MAE and RMSE to measure the effectiveness of the model and quantify the computational complexity based on the number of network parameters (M) and the number of floating-point operations per second (G). In the table header, a downward arrow signifies that a smaller value indicates better performance.

**Table 7:** A comparison of the computational complexity of different methods.

Methods	AVEC2013		AVEC2014		Param.↓	FLOPS↓
	RMSE↓	MAE↓	RMSE↓	MAE↓		
ResNet-50 [11]	8.81	6.92	8.40	6.79	63	12.22
TS-RNN + 3DCNN [10]	8.28	7.37	9.22	7.20	33.64	8.51
MDN-50 [11]	8.13	6.39	8.16	6.45	21	7.40
MSN [51]	7.90	**5.98**	**7.61**	**5.82**	77.70	164.90
MTDAN [22]	8.08	6.14	7.93	6.35	**0.90**	**1.97**
OpticalDR [52]	8.48	7.53	8.82	7.89	67.28	4.36
Ours	**7.83**	6.48	7.82	6.30	5.08	2.85

In our table, we use bold and underline to highlight the best and second-best results. In the table header, a downward arrow signifies that a smaller value indicates better performance.

**Table 8:** Robustness analysis of models using different methods.

Dataset	Robustness Testing Methods	RMSE ↓	MAE ↓
AVEC2013	Image Rotation by 30 Degrees	8.63	6.96
	Image Rotation by 60 Degrees	8.95	7.03
	Image Rotation by 90 Degrees	8.67	6.98
	Image with Gaussian Noise	9.35	7.28
	Image with Random Noise	8.49	7.03
	Image with Random Occlusion	8.11	6.86
AVEC2014	Image Rotation by 30 Degrees	8.72	6.96
	Image Rotation by 60 Degrees	8.90	7.08
	Image Rotation by 90 Degrees	8.74	7.01
	Image with Gaussian Noise	9.12	7.33
	Image with Random Noise	8.26	6.75
	Image with Random Occlusion	8.12	6.57

In the table header, a downward arrow signifies that a smaller value indicates better performance.

## Data Availability

Data are available on request.

## Abbreviations

The following abbreviations are used in this manuscript:

TCEDN	Time-Context Enhanced Depression Detection Network
3D-CNN	3-Dimensional Convolutional Neural Network
ConvLSTM	Convolutional Long Short-Term Memory Network
AVEC2013	Audio/Visual Emotion Challenge 2013
AVEC2014	Audio/Visual Emotion Challenge 2014
MDD	Major Depressive Disorder
HAMD	Hamilton Depression Rating Scale
BDI-II	Beck Depression Inventory-II
PHQ-9	Patient Health Questionnaire-9
CNNs	Convolutional Neural Networks
LSTM	Long Short-Term Memory
AWAM	Attention-Weighted Aggregation Module
RNN	Recurrent Neural Network
2D-CNN	2-Dimensional Convolutional Neural Network
LPQ	Local Phase Quantization
SVR	Support Vector Regression
MHH	Motion History Histogram
LGBP-TOP	Local Gabor Binary Patterns from Three Orthogonal Planes
LBP-TOP	Local Binary Patterns from Three Orthogonal Planes
LPQ-TOP	Local Phase Quantization from Three Orthogonal Planes
FDHH	Feature Dynamic History Histogram
DLGA-CNN	Deep Local Global Attention CNN
DAERNet	Dual Attention and Element Recalibration Network
STDWM	Self-Learning Time-Difference Weighting Module
SWFM	Self-Learning Weight Fusion Module
MTCNN	Multi-Task Cascaded Convolutional Network
MAE	Mean Absolute Error
RMSE	Root Mean Square Error
PLS	Partial Least Square
MHI	Motion History Image
MFA	Marginal Fisher Analysis
DTL	Deep Transformation Learning
DepressNet	Deep Regression Networks
MSN	Multi-scale Spatiotemporal Network
MAD	Maximization and Differentiation Network
LQGDNet	Local Quaternion and Global Deep Network
MTDAN	Multi-scale Temporal difference Attention Networks
OpticalDR	Optical Imaging Model for Privacy-Protective Depression Recognition
rPPG	remote photoplethysmography signals
Grad-CAM	Gradient-Weighted Class Activation Mapping
